# Structural and Parametric Identification of Knowm Memristors

**DOI:** 10.3390/nano12010063

**Published:** 2021-12-27

**Authors:** Valerii Ostrovskii, Petr Fedoseev, Yulia Bobrova, Denis Butusov

**Affiliations:** 1 Department of Computer-Aided Design, St. Petersburg Electrotechnical University “LETI”, 197376 Saint Petersburg, Russia; psfedoseev@etu.ru; 2 Department of Biomedical Engineering, St. Petersburg Electrotechnical University “LETI”, 197376 Saint Petersburg, Russia; jobobrova@etu.ru; 3 Youth Research Institute, Saint Petersburg Electrotechnical University “LETI”, 197376 Saint Petersburg, Russia

**Keywords:** memristor, identification, voltage-current curve, memristive device, nonlinear component

## Abstract

This paper proposes a novel identification method for memristive devices using Knowm memristors as an example. The suggested identification method is presented as a generalized process for a wide range of memristive elements. An experimental setup was created to obtain a set of intrinsic I–V curves for Knowm memristors. Using the acquired measurements data and proposed identification technique, we developed a new mathematical model that considers low-current effects and cycle-to-cycle variability. The process of parametric identification for the proposed model is described. The obtained memristor model represents the switching threshold as a function of the state variables vector, making it possible to account for snapforward or snapback effects, frequency properties, and switching variability. Several tools for the visual presentation of the identification results are considered, and some limitations of the proposed model are discussed.

## 1. Introduction

The fourth fundamental two-terminal passive circuit element, the memristor, was postulated by L.O. Chua in 1971 based on the principle of symmetry between electrical quantities [1]. Significant attention to this topic was drawn after its association with TiO_2_-based resistive switching devices by HP Labs in 2008 [2]. Since then, any devices which exhibit specific properties, named “the fingerprints” [3], are usually referred to as memristors. Promising applications of memristive devices include non-volatile memory [4], logic circuits [5], sensing [6], cryptography [7], chaotic generators [8], and neuromorphic computing [9]. Development of the latter direction is performed from the standpoint of using memristors as synaptic connections in artificial neural networks [10], mimicking biological architectures in the nervous systems. The most recent progress in the study of memristors in bio-inspired circuits was made in [11,12].

In the simplest case, memristive devices are represented by a metal-insulator-metal (MIM) structure, whose conductivity varies depending on external voltage or current. Many materials have been reported to be suitable for creating memristors [13], wherein resistive switching mechanisms are classified according to a manifestation of different memory effects [14]. The HP memristors with bipolar switching are oxygen-ion conducting cells with valence change memory (VCM) effect. Significant nonlinearities in ionic transport, responsible for the hysteresis behavior of a TiO_2_ memristor, arise by reaching nanoscales [2]. The creation of such devices requires high-level nanotechnology and advanced equipment. At the same time, the study of the nonlinear properties of memristors also requires expert knowledge in the field of dynamical systems, where researchers do not always have access to thin-film fabrication technology. Currently, this gap is filled with commercially available devices, among which are the memristors distributed by Knowm Inc.

The operation principle of Knowm memristors is similar to HP memristors and is based on the redox phenomena. However, Knowm devices make use of the electrochemical metallization (ECM) memory effect and therefore belong to a different branch of memristive cell taxonomy, forming a separate class of self-directed channel (SDC) memristors [15]. Since 2017, several studies on systems with SDC memristors have been published. An attempt at modeling Knowm devices by selecting parameters of generic memristor models was made by B. Garda and Z. Galias in 2018 [16]. The best approximation was obtained from a voltage threshold adaptive memristor model (VTEAM [17]); however, the fitting error was significant even in this case. An interesting paper [18] presented and evaluated accessible experimental measurement setups for memristors on the example of the Knowm devices, but modeling issues were not considered. Drake et al. [19] and Bunnam et al. [20] discussed the temperature characteristics of SDC memristors. In [19], the research group affiliated with Knowm Inc. provided some experimental results for devices that differ in structure from commercial devices. In [20], the characteristics of Knowm devices are shown, a model of temperature dependence is proposed suggesting similarity to the TiO_2_ memristors exponential dependence, but the memristivity function was not specified. In 2020, the first reports of chaotic circuits with Knowm memristors appeared. In [21,22], C.K. Volos et al. demonstrated chaotic modes of Shinriki’s circuit [23] modified by adding an SDC memristor. Despite studying the circuit equations, the complete memristive device model was not presented in these works. In [24], Minati et al. adapted Sprott’s jerk circuit [25] to exploit nonlinearities of SDC memristors for the appearance of chaotic attractors. In order to explain the observed dynamics, the authors applied the mean metastable switch (MMS) model of a memristor, recommended by the Knowm Inc. affiliated researchers [26,27].

In the Knowm SDC memristors datasheet [28] and the abovementioned research works, all of the given I-V curves are shown at rather high currents in the range of 10^−4^–10^−2^ A. Meanwhile, the manufacturer strongly recommends limiting the current with a 50 kΩ series resistor at regular device operation under 1 V (maximum allowable voltage range of −5–3 V). Thus, the comprehensive investigation of the SDC memristors under low-current (less than 10^−5^ A) operation is still in demand being a key to operational safety and energy efficiency of memristor-based systems. In this paper, we will explicitly show that the MMS memristor model cannot capture all of the significant switching properties of the Knowm devices in such operating conditions. Considering also the variability of memristive devices (see, e.g., [29]), it is of interest to create a method for constructing new memristor models.

In [30], A. Fantini et al. investigated switching characteristics of HfO_2_ devices in the low current operation regime. This work paid particular attention to the voltage snapback effect for which to describe a quantum mechanical model was proposed. In [31], the research was extended to Al_2_O_3_ devices, and the conductance quantization description was refined as a quantum point-contact model. D. Niraula and V. Karpov in [32] proposed a comprehensive model, adopted for the low-current snapforward and snapback effects, as well as cycle-to-cycle switching variability, which was represented as particle dynamics in a finite number of double-well potentials. Unfortunately, operating with the description of processes in partial derivatives, these models are quite complex and not suitable for use in software circuit simulation environments. A much simpler, SPICE-suitable phenomenological model for the snapback effect in memristive devices was proposed by E. Miranda et al. [33]. The model parameters were selected for the Ta_2_O_5_-based structure. The disadvantages of this model follow from its discrete nature, the deviation from the concept of analog memristor, and the lack of accounting for cycle-to-cycle variability. Thus, we conclude that there is a strong need for compact models allowing adequate simulation of memristors in low-current switching regimes.

In this paper, we propose the identification method for memristive devices and a new Knowm memristor model that considers low-current effects and cycle-to-cycle variability. For the first time, the development principles of new chaotic memristor models, approximating the behavior of real devices, have been formalized, which determines the scientific novelty of the proposed identification method. The main contributions of the study are as follows:

1. The novel identification method is presented as a generalized process for a wide range of memristive elements.
2. The proposed memristor model outperforms the existing ones in representing the switching threshold as a function of the state variables vector, making it possible to account for snapforward or snapback effects, frequency properties, and switching variability.
3. The process and results of the parametric identification for the proposed memristor model are presented.

The rest of the paper is organized as follows. In Section 2, the identification method, the structure of the investigated device, an experimental setup, modeling criteria, and candidate models are presented. In Section 3, the limitations of the selected models are considered, the modified model is proposed, and the stages of parametric identification are demonstrated. In Section 4, some tools for visual presentation of identification results are considered and the limitations of the proposed model are discussed. Finally, Section 5 concludes the paper.

## 2. Materials and Methods

### 2.1. Procedure for Identification of Memristive Elements

**Figure 1:**
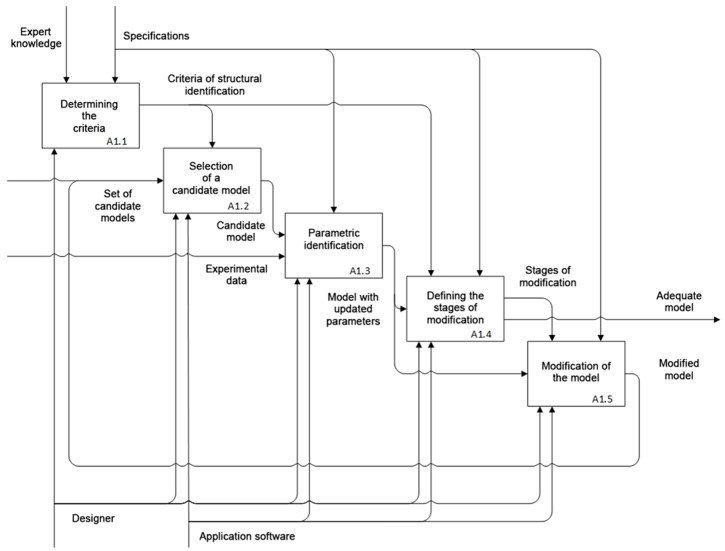
Process of memristive element identification.

The identification procedure involves the experimental study of the object and the comparison of its input and output. Thus, the identification problem assumes synthesizing (or selecting from the group of available ones) an adequate mathematical model for the object. The proposed technique for the identification of memristive elements includes the stages of structural and parametric identification. IDEF0 diagram of the identification process is shown in Figure 1.

The input of the presented identification process is experimental data and a set of candidate models. The choice of the general structure of a memristor model and the class of equations describing the switching processes is carried out at A1.2, one of the stages of structural identification (processes A1.1, A1.2, A1.4, and A1.5). In order to solve the problem of structural identification, it is required to use a priori information on the dynamics of resistive switching in memristors; the development of criteria is carried out at stage A1.1 based on this knowledge. The adoption of design decisions on modifying the most suitable model (process A1.4) is carried out after selecting the optimal model parameters concerning the experimental data at the stage of parametric identification A1.3. If modification is not required, then the selected model with the opted parameters is considered adequate and is fed to the output of the identification process. Otherwise, research is required to modify the mathematical model and add it to the set of candidate models, organizing feedback within the identification process. In parametric identification A1.3, tasks of the experimental data utilization are solved specific to the selected memristor model. Problems regarding the conditions for data acquisition are carried out at the preliminary to identification stages.

### 2.2. Knowm Memristive Devices

**Figure 2:**
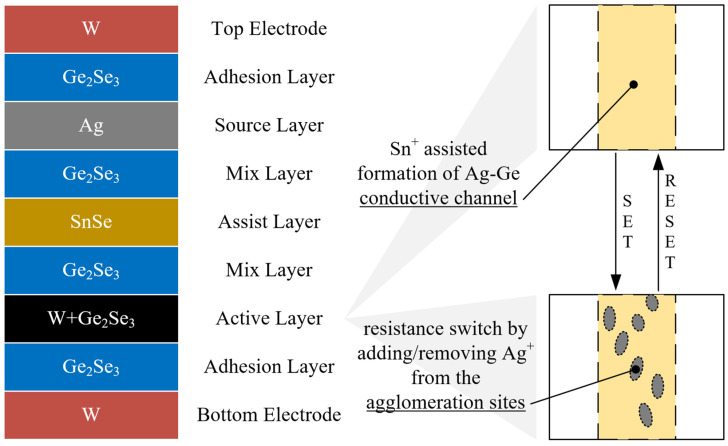
Stack of W-dopant Knowm memristor. materials (**left**) and a graphical representation of the switching mechanism (**right**). Reprinted from Ref [28].

Knowm Inc. produces four versions of memristors [28]: W, C, Sn, and Cr, according to the dopant introduced into the active layer during fabrication. Each dopant changes the dynamical switching characteristics of a device. Figure 2 presents the W memristor device structure.

The datasheet on Knowm memristors [28] provides the following description of resistive switching. During the forming process, which requires the application of an appropriate positive potential to the top electrode, Sn ions from the assist SnSe layer enter the active Ge_2_Se_3_ layer, where they facilitate the substitute ion of Ge on the Ge-Ge bond for Ag ions from the source layer. Areas where Ge-Ge dimers turn into Ag-Ge bonding sites form the self-directed channel. Since Ag has a tendency to agglomerate with other Ag atoms, the Ag-Ge sites constitute conductive clusters. By applying either a positive or negative potential across the device, one can vary concentrations of Ag within the clusters, establishing the mechanism for resistive switching.

### 2.3. Experimental Setup

**Figure 3:**
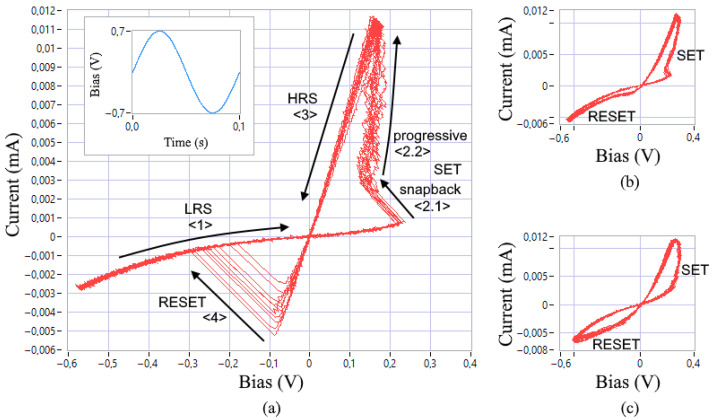
Experimental I-V curves of devices with the property of resistive switching, examples (**a**–**c**) were obtained after forming the devices in different conditions.

The proposed identification method is based on experimental data on I-V curves of the investigated memristive devices. The IV curves were obtained using NI ELVIS III data acquisition equipment. In order to limit device current, the Knowm manufacturer recommends the utilization of a series resistor and suggests taking $R_{s}$ = 50 kΩ [28]. A voltage divider circuit with a resistor $R_{s}$ = 46.25 kΩ placed in series was applied to measure the I-V curve, which is sufficient for low-current memristor operation with the voltage snapback effect. Figure 3 shows 10 obtained I-V curve cycles of the Knowm memristor with W dopant under a sinusoidal control voltage at a frequency of 10 Hz with an amplitude of 0.7 V. Figure 3a denotes the boundaries of the high resistive state (HRS) and the low resistive state (LRS), the I-V curve direction in the Section 1, Section 2, Section 3 and Section 4 the SET and RESET switching processes, which are also indicated in subfigures (b) and (c).

### 2.4. Modeling Criteria

In [34], E. Linn et al. formulated three criteria for assessing the mathematical modeling adequacy for the redox-based bipolar resistive switching devices:

1 Correspondence of the model’s I-V curve and the switching dynamics in the time domain to the experimental data of real devices. Therefore, in Figure 3a,b, the SET transition process of the device from HRS to LRS demonstrates a sharp current increase with a shift of the voltage switching boundary (snapback) at the initial stage <1>, in case (c) the SET transition looks smooth throughout the entire section. The reverse RESET process, which switches the device to HRS, can either be instantaneous (a), snapforward effect, or significantly slower (b) and (c). The symmetry of the I-V curve relative to the diagonal of the II and IV quarters is often violated. This can also be visualized in the time domain when AC voltage is applied. In addition, there is a visible curvature of the <1> section due to the metal-semiconductor/insulator barrier between the electrodes and the inner layers of the devices.
2 Nonlinearity of the switching function. The origin of this nonlinearity in memristive devices based on redox reactions is explained by the nonlinear movement of ion vacancies or defects, accelerated by Joule heating. This property is characterized in that the resistance switching time of the SET and RESET processes decreases by orders of magnitude if the applied voltage pulse increases only several times. Thus, this criterion tests the model for a nonlinear dependence of the switching time on the input voltage.
3 Suitability for modeling the complementary serial connection of two elements. One of the distinguishing features of such a connection is the presence of a common LRS when AC voltage is applied. This criterion serves as a check for the consistency of the memristive device model.

Later in [35], S. Menzel et al. expanded the list of criteria to six, including dynamical properties of VCM devices:

4 Ability to set several states of resistance. The criterion is to identify more than two states of resistance of the memristive device between the LRS and HRS, providing multi-bit data storage.
5 Dependence of SET (or RESET) switching from the current state of the resistance. According to this criterion, the voltage required to set the device to a lower resistance state should depend on the high resistance value in the current cycle and vice versa. Thus, the switching kinetics should be power-dependent.
6 Reliable simulation of the memory fading effect. This dynamical phenomenon is well known in the theory of nonlinear systems. With suitable periodic exposure, the previous “history” of the memristive device is gradually erased.

Let us include the above criteria in the method for the identification of memristive elements, supplementing the list with a requirement targeted on the compliance of models of memristive devices with the technical limitations of computer simulation and hardware execution within the framework of the research-based design methodology:

7 Compact representation of a continuous mathematical model, which determines the suitability of using its discrete version in the processes of large-system simulation, including neural networks, as well as digital hardware emulators of memristive circuits.

### 2.5. Candidate Memristor Models

#### 2.5.1. Mean Metastable Switch Memristor Model

Knowm researchers adhere to the concept of AhaH computing [36], in which M. Nugent and T. Molter presented a semi-empirical model of metastable memristor switching to describe the transition from stochastic binary to incremental analog properties.

Metastable switching is an idealized element with two states corresponding to high and low resistance, between which it switches with different probabilities depending on the applied voltage and temperature. The probability of such an element moving from LRS to HRS is defined as *P_OFF_*, and the probability that a switch will change from HRS to LRS is defined as *P_ON_*. A metastable switch memristor can be represented as a set of *N* switching elements with dynamical evolution over discrete time intervals. The mean metastable switch memristor (MMS, [27]) model implements the limiting case when N→∞.

The change in the number of switches *X*, scaled from 0 to 1, is defined as:

(1) $$dX=N_{ON}-N_{OFF},$$

where the number of switches is represented as:

(2) $$\begin{array}{c} N_{ON}=P_{ON}(1-X)\\ N_{OFF}=P_{OFF}X. \end{array}$$

Switching probabilities when voltage *V* is applied are given as:

(3) $$\begin{array}{c} P_{ON}=\alpha \frac{1}{1+e^{-\beta (V-V_{ON})}}\\ P_{OFF}=\alpha \left(1-\frac{1}{1+e^{-\beta (V+V_{OFF})}}\right), \end{array}$$

where *V_ON_* is the threshold voltage for switching to the low resistance state, *V_OFF_* is the threshold voltage for switching to the high resistance state, $\beta =q/kT=V_{T}^{-1}\;$ is the temperature parameter, *q* is the elementary charge, *k* is the Boltzmann constant, *T* is the absolute temperature, α=dt/τ is the time parameter, and *τ* is the time constant of the memristor.

Then, the equation of the state variable of the memristor takes the form of:

(4) $$\frac{dX}{dt}=\frac{1}{\tau }\left(\frac{1}{1+e^{-\beta (V-V_{ON})}}(1-X)-\left(1-\frac{1}{1+e^{-\beta (V+V_{OFF})}}\right)X\right),$$

furthermore, the conductivity of the memristor is as follows:

(5) $$G=\frac{X}{R_{ON}}+\frac{1-X}{R_{OFF}}.$$

This model was used by Minati et al. in [24] to describe the dynamics of a memristor switching as part of a chaotic circuit.

#### 2.5.2. Generalized Mean Metastable Switch Memristor Model

In the generalized mean metastable switch memristor (GMMS, [27]) model, the total current through the device is represented by the sum of the currents of the resistive memory element *I_M_*(*V*, *t*) (MMS model) and the Schottky diode *I_S_*(*V*):

(6) $$I=\phi I_{M}(V,t)+(1-\phi )I_{S}(V),$$

where ϕ∈[0,1]. A value of *ϕ* = 1 implies that there is no Schottky diode current.

The *I_S_*(*V*) current is required to represent the Schottky barrier over the metal-semiconductor junction, and in turn, can be decomposed into forward and reverse bias components as follows:

(7) $$I_{S}=\alpha_{f}e^{\beta_{f}V}-\alpha_{r}e^{\beta_{r}V},$$

where *α_f_*_,*r*_ and *β_f_*_,*r*_ are positive parameters that specify the exponential behavior of the forward and reverse current flowing through the Schottky barrier.

## 3. Results

### 3.1. Criterial Analysis of Candidate Models

As a part of structural identification of the memristive element, it is necessary to establish the static and dynamic components of the model. Using static (algebraic and/or transcendental) equations, the relationship between the electron and ion currents, as well as the resistance/conductivity function of the element, are determined. The dynamic component in the form of an ODE system must determine the internal state variables and their corresponding right-hand side functions. The key process in structural identification, as is shown in Figure 1, is process A1.4 which defines the stages of modification for the most suitable model from the set of candidates, and process A1.5 performs the modification to achieve the unmet criteria of the candidate model. These processes are preceded by choice of criteria (given at the beginning of the article) and the initial model, as well as the process of parametric identification to determine the characteristics of the model under conditions approximate to the experiment. When the stages of modification are not determined during structural identification due to the satisfaction of all the criteria, the model is considered adequate to the design object in the operating mode of interest.

**Figure 4:**
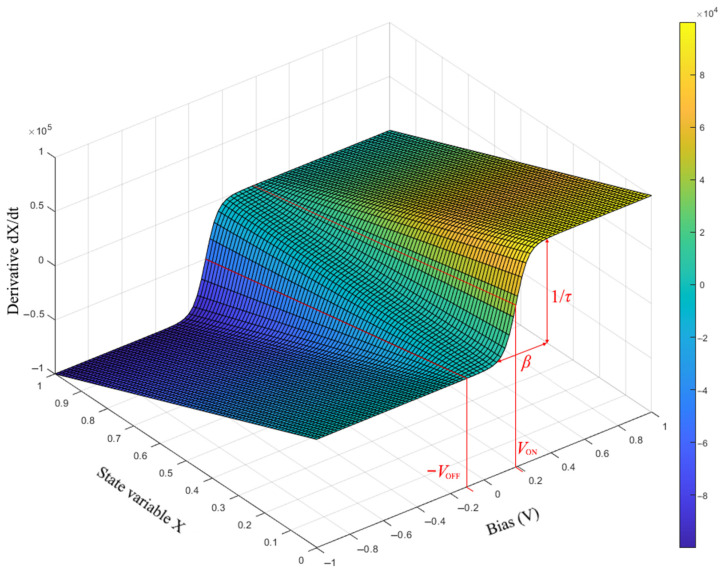
Surface represents the function of the right-hand side of Equation (4) of the MMS model.

In this study, we use the MMS model as the initial candidate model for Knowm devices. This model belongs to the class of models with voltage thresholds *V*_ON_ and *V*_OFF_. Like the similar mem-diode model [37] (whose equations are much more complicated to analyze), the MMS model uses the sigmoid switching function of the internal state variable *X*. Figure 4 shows the surface corresponding to the function of the right-hand side *dX/dt*(*X*, *V*). The β parameter in Equation (4) is responsible for the width of the sigmoid, 1/*τ* determines the maximum rise height along the sigmoid, the *V*_ON_ and *V*_OFF_ parameters set the coordinate of the center point of the sigmoid along the voltage axis *V*.

**Figure 5:**
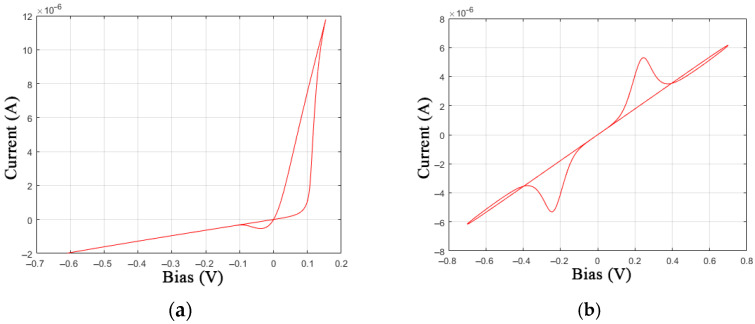
I-V curve of the MMS model: (**a**) a single element; (**b**) a complementary series connection of two elements.

The MMS model in its basic form satisfies criteria 2, 3, 4, 6, and 7, which we highlighted in Section 2.4. One can see that Figure 5a shows the simulated MMS (*R*_ON_ = 5000 Ω, *R*_OFF_ = 10^5^ Ω, *V*_ON_ = 0.2 V, *V*_OFF_ = 0.1 V, *τ* = 0.0001, *T* = 298.5 K) I-V curve of a single memristor under conditions similar to the experiment in Figure 3.

According to criterion 2, the nonlinearity of the switching function is implemented by Equation (4) in the form of a sigmoid, as shown in Figure 4, and contains a temperature dependence in the parameter *β = q/kT*. The compliance with criterion 3 is illustrated in Figure 5b, where one can see the appearance of a threshold value for switching from LRS to HRS with increasing voltage, which is typical for a complementary series connection of two elements. The atypical loops between (−0.8, −0.4) and (0.4, 0.8) voltage intervals in Figure 5b are caused by the inequality of the parameters *V*_ON_ ≠ *V*_OFF_.

**Figure 6:**
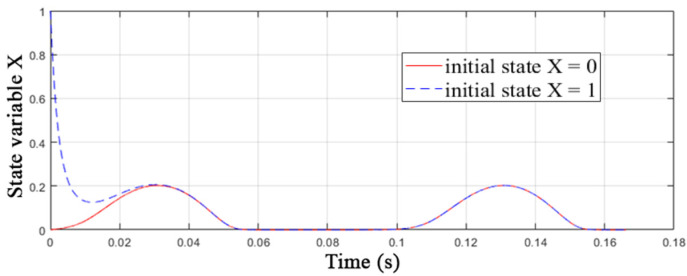
Effect of memory fading and intermediate states of the MMS model.

Criterion 4 is also met due to the possibility of setting the state variable *X*, which is responsible for the conductivity of the element, intermediate values between 0 and 1, as shown in Figure 6. In order to demonstrate the fulfillment of criterion 6, the process of two identical memristors simulation was launched with the boundary initial conditions of the internal state variable *X*(0) = 0 and *X*(0) = 1, a sinusoidal control voltage with an amplitude of 0.1 V and a frequency of 10 Hz was applied. As shown in Figure 6, the coincidence of the time series is achieved already in the first switching cycle, which indicates the effect of memory fading in the MMS model.

Finally, it is worth noting the simplicity of Equations (4) and (5) of the MMS model, compared to the universal models VTEAM [17] and Stanford [38], belonging to the class of compact ones, which states that criterion 7 is satisfied.

Now, we will consider criteria 1 and 5, which the MMS model does not meet. The differences between the experimental and simulated I-V curves can be seen by comparing Figure 3a and Figure 5a. One can see that criterion 5 cannot be met because of the fixed switching thresholds *V*_ON_ and *V*_OFF_.

**Figure 7:**
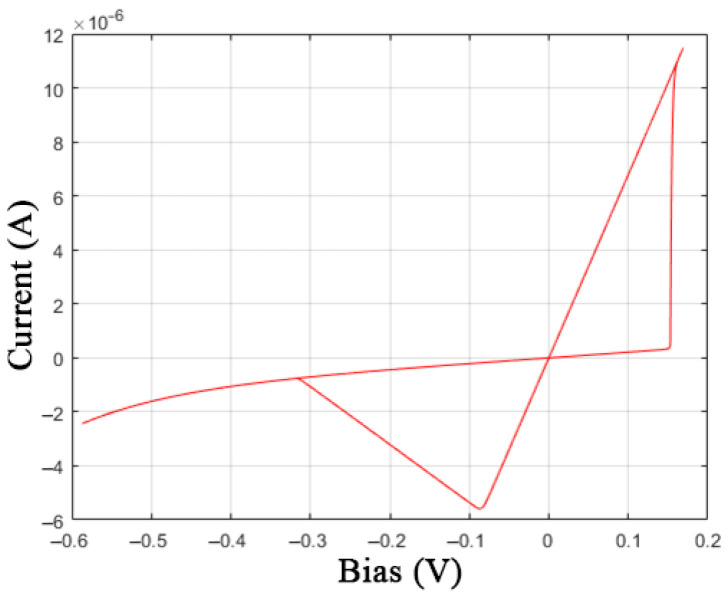
I-V curve of the GMMS model of the memristive element.

Additional equations of the GMMS model make it possible to bring the simulated I-V curve closer to the experimental data by adding bends in the Section 1 and Section 3, marked in Figure 3a. The closest fit (Figure 7) was obtained when choosing the parameters: *R*_ON_ = 13,000 Ω, *R*_OFF_ = 4.6·10^5^ Ω, *V*_ON_ = 0.17 V, *V*_OFF_ = 0.1 V, *τ* = 6·10^−5^, *T* = 28.5 K, *ϕ* = 0.88, *α*_f_ = *α*_r_ = 10^−7^, and *β*_f_ = *β*_r_ = 8. In this case, for an expressed acceleration of switching processes, it was necessary to reduce the memristor time constant *τ* by order of magnitude and the temperature parameter *T* to a value with no physical meaning, which may be associated with the observation of quantum effects.

### 3.2. The Modification of Memristor Model

**Figure 8:**
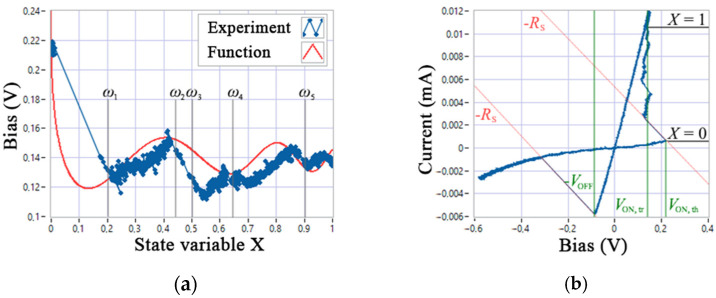
Experimental data: (**a**) dependence of the threshold voltage on the internal state variable *X* during SET; (**b**) I-V curve of one cycle with a length of 1 s.

Further modification of the GMMS model targets achieving criteria 1 and 5 and can be performed as follows. At the first stage, it is necessary to introduce a functional dependence of the threshold parameters *V*_ON_ and *V*_OFF_ on the internal state variable *X*, which is associated with the appearance of a section of negative differential resistance of the I-V curve in the first quarter when using a measuring circuit with a voltage divider. An array of experimental data of voltage depending on the variable *X* when switching SET is shown in Figure 8a, where one can see the snapbacks, i.e., abrupt current jumps with a slope inversely proportional to the load *R*_S_ = 46.25 kΩ. A series-connected resistor *R*_S_ allows stabilizing the LRS and the switching process of the SET device (the effects were studied in [30,39,40]), as well as revealing the fact that the *V*_ON_ switching boundary has shifted from 0.22 V (*V*_ON,th_) to values less than 0.12 V in average (*V*_ON,tr_) close to *V*_OFF_~0.1 V (see Figure 8b).

Abrupt jumps in the I-V curves in switching processes can be explained by a discrete change in the quantum states of the system (see a quantum mechanical switching model described in [41]) with a change in the ion concentration in the active layer of the device, when the motion of the atoms of the conducting channel has a significant effect on small currents. In Figure 8a, the states ω 1–5 are marked for the SET process; note that their approximation by a piecewise-specified function on the entire interval [0, 1] has no practical meaning, since at low currents (under experimental conditions <12 μA), only the first state *ω*_1_ is stable, as can be seen in Figure 3a,b. This observation necessitates modeling only the first jump, which can be performed using a continuous function, which in the conditions of this experiment (Figure 8a) takes the form:

(8) $$V_{ON}(X)=\frac{0.1\cos\left(\left(4\pi \sqrt{X}\right)/\left(1.7-X\right)\right)}{1+10\sqrt{X}}+0.14.$$

**Figure 9:**
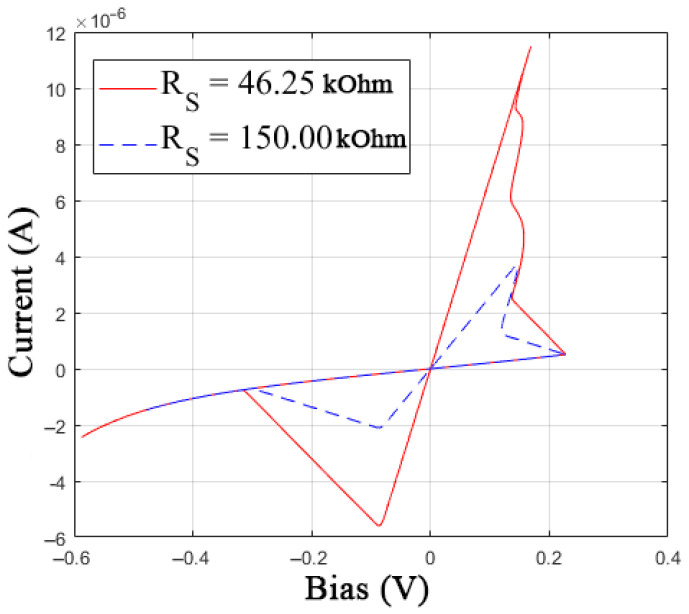
I-V curve of the modified model of GMMS memristive element with function (8).

The I-V curves of the GMMS model with the *V_ON_*(*X*) function are shown in Figure 9. Depending on *R*_S_, the slope of the SET switching line changes correctly.

The proposed modification of the GMMS model makes it possible to fulfill the conditions of criterion 5 for the experimentally observed operating mode of the investigated device. When considering a single switching cycle, the model also satisfies criterion 1.

**Figure 10:**
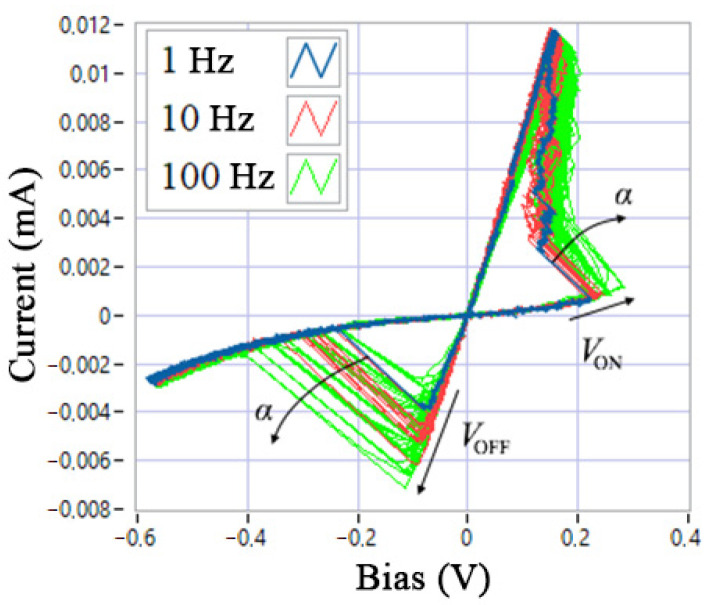
Experimental I-V curve of the device at different frequencies of the sinusoidal control voltage.

The second stage of structural identification is aimed at frequency modification of the memristor model. Figure 10 shows the experimentally obtained I-V curve of the device under study at frequencies 1, 10, and 100 Hz. As the frequency increases, the average switching threshold values *V*_ON_ and *V*_OFF_, as well as the deviation of the angles of the breaking lines *α* from the load value *R*_S_, increase.

The increase in the angle α is correctly predicted by the GMMS model with the correct selection of the time constant *τ*. An estimate of the control signal frequency may be necessary to represent the offset of the *V*_ON_ and *V*_OFF_ boundaries adequately. The frequency *f* of the triangular voltage control signal is determined by the first derivative *dV/dt*, sinusoidal—the second derivative (from the equation d^2^*V*/d*t*^2^ + *f*^2^*V* = 0). In a measuring circuit based on a voltage divider, the memristor voltage is cut off in the SET process with the appearance of a negative differential voltage section, which complicates the static calculation of the frequency. In this case, one can use the dynamic estimation of the frequency *F*:

(9) $$\frac{dF}{dt}=a_{F}\left(\sqrt{\left|\frac{d^{2}V}{dt^{2}}/V\right|}-b_{F}F\right),$$

where *a_F_* is the time coefficient of the state variable *F*, *b_F_* is the feedback coefficient, *V* is the voltage across the memristor.

When considering typical electrical circuits with memristive elements, the function d^2^*V*/d*t*^2^ can often be derived analytically. However, in the general case, it requires numerical differentiation using finite difference methods.

**Figure 11:**
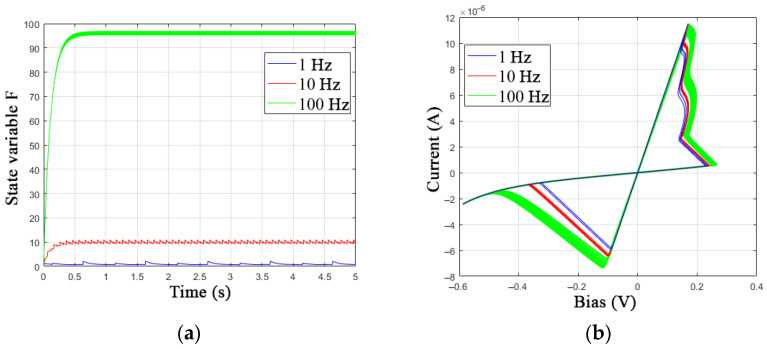
Modification of the GMMS model: (**a**) the values of the state variable F and (**b**) the I-V curve of the memristive element at different frequencies of the sinusoidal control voltage.

Figure 11 shows an example of the operation of the proposed modified memristor model with the addition of additive terms in *V*_ON_ and *V*_OFF_ from the state variable *F* at *a_F_* = 10 and *b_F_* = 0.9. As the signal frequency increases, one can see an increase in the angle *α* and the voltage thresholds *V*_ON_ and *V*_OFF_.

The third stage of structural identification involves the final modification of the model, taking into account the variability of the SET and RESET switching processes, as shown in Figure 3 and Figure 10.

The most common approach is considering the variability of switching as a manifestation of stochastic processes. Stochastic models of memristive elements, created by adding, e.g., white Gaussian noise, are proposed by N.V. Agudov [42,43]. Some other methods for synthesizing stochastic models of threshold-type memristors are also well described in [44,45].

An alternative approach to reproducing fluctuations in switching processes is chaotic dynamics. The key advantage of this approach is that the model remains deterministic, which simplifies the stages of verification and testing of memristive circuits during their design. In their recent paper [46], Driscoll et al. considered the chaotic Duffing oscillator as a memristive model, while the physical substantiation is the connection of the equations of the oscillator with the dynamics of particles in potential wells of nanoscale devices [32]. We will use the technique of embedding a chaotic generator into a regular memristor model to introduce variability into the threshold voltage values *V*_ON_ and *V*_OFF_. The Duffing oscillator equations are as follows:

(10) $$\begin{array}{l} \frac{dY}{dt}=a_{Y}Z\\ \frac{dZ}{dt}=a_{Z}\left(c_{Z}S-b_{Z}Z+Y-Y^{3}\right), \end{array}$$

where *Y* and *Z* are state variables, *S* is an external signal, *a_Y_* and *a_Z_* are time coefficients of the system, *b_Z_* is a feedback coefficient, and *c_Z_* is a signal coefficient.

Thus, the final system of equations for the modified model of the GMMS memristor at a harmonic input voltage takes the form of:

(11) $$\begin{array}{l} I=\phi I_{M}(V,t)+(1-\phi )I_{S}(V)\\ I_{S}=\alpha_{f}e^{\beta_{f}V}-\alpha_{r}e^{\beta_{r}V}\\ I_{M}=\left(\frac{X}{R_{ON}}+\frac{1-X}{R_{OFF}}\right)V\\ \frac{dX}{dt}=\frac{1}{\tau }\left(\frac{1}{1+e^{-\beta (V-V_{ON}(X,F,Y,Z))}}(1-X)-\left(1-\frac{1}{1+e^{-\beta (V+V_{OFF}(X,F,Y,Z))}}\right)X\right)\\ \frac{dF}{dt}=f\left(F,V,\frac{dV}{dt},\frac{d^{2}V}{dt^{2}}\right)\\ \frac{dY}{dt}=a_{Y}Z\\ \frac{dZ}{dt}=a_{Z}\left(c_{Z}S-b_{Z}Z+Y-Y^{3}\right). \end{array}$$

**Figure 12:**
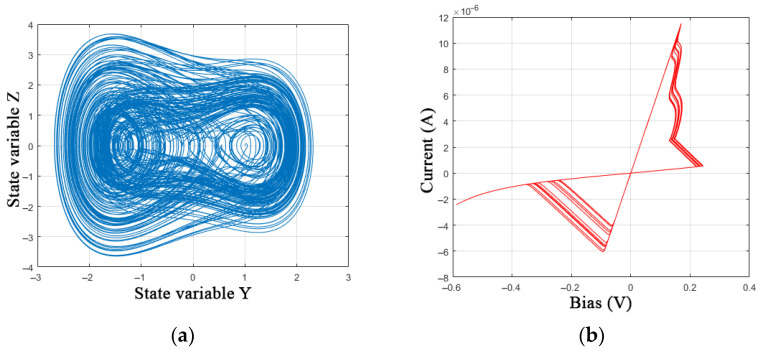
Modification of the GMMS model: (**a**) phase portrait of a chaotic generator and (**b**) I-V characteristic of a memristive element at a frequency of 10 Hz.

The voltage across the memristor *V* can be used as *S*; however, from a practical point of view, chaotic modes are better expressed at *S* = cos(*Ft*). Figure 12 shows an example of the chaotic model of the GMMS of a memristive element.

Next, let us consider the procedure for selecting the parameters of the modified model according to the experimental data of the Knowm memristor operation in the mode shown in Figure 3a.

### 3.3. Parametric Identification

**Figure 13:**
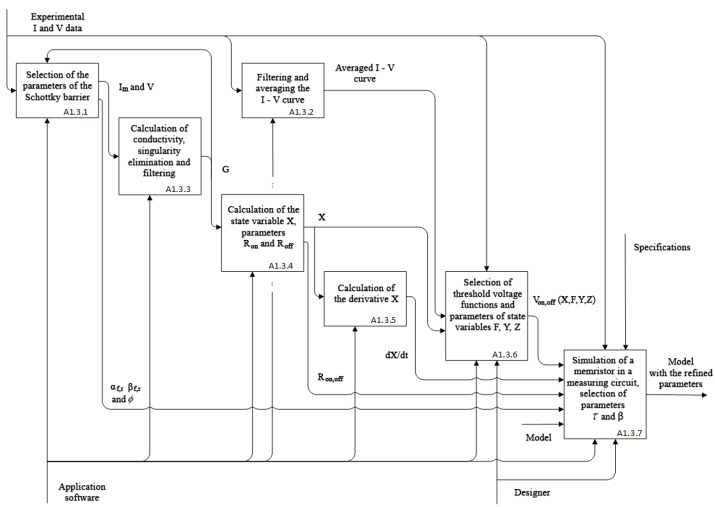
Process of parametric identification of memristive elements.

The process of parametric identification of the memristor model is presented in the IDEF0 diagram in Figure 13. The execution of stages that involve only the use of applied software can be performed automatically by existing methods. At the same time, the participation of designers is required at two final stages of the parametric identification process: in A.1.3.6 for solving the problem of adapting a chaotic generator, in A1.3.7 for evaluating the technical characteristics of a discrete model.

**Figure 14:**
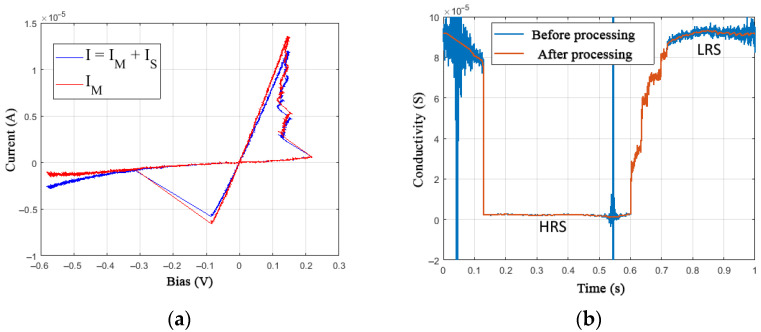
(**a**) Experimental I-V curves of the device at a frequency of 1 Hz when selecting the parameters of the Schottky barrier and (**b**) processing the conductivity data.

In the cycle of stages A1.3.1 and A1.3.3 of parametric identification, the values of the parameters of the Schottky barrier *α*_f,r_, *β*_f,r_, and *ϕ* from Equations (6) and (7) are determined based on the experimental data by reducing the value of the derivative calculated from the current *I*_M_ of conductivity dG/dt to a minimum in the HRS section, which is much less noisy in comparison with the LRS (Figure 14b). The selection of optimal parameters leads to the rectification of the HRS and LRS sections on the I-V curve (Figure 14a) when displaying the current *I*_M_.

The processing of the conductivity data assumes filtering, as well as the elimination of the singularity regions caused by dividing by close to zero voltage values *V*. To determine the switching dynamics, which is associated with the operation of numerical differentiation, it is essential to preserve the actual conductivity values from the experiment, which imposes additional restrictions on the filter being used. In the example shown in Figure 14b, data filtering was applied on the HRS and LRS sections.

At stage A1.3.4, the parameters *R*_ON_ and *R*_OFF_ are determined using the Formula (5). The state variable *X* is calculated by normalizing the processed conductivity data at a frequency of 1 Hz, at which achieving the maximum and minimum memristor resistance is guaranteed. When calculating the state variable *X* from experimental data at high frequencies, it is necessary to use the values of the *R*_ON_ and *R*_OFF_ parameters determined at a frequency of 1 Hz.

**Figure 15:**
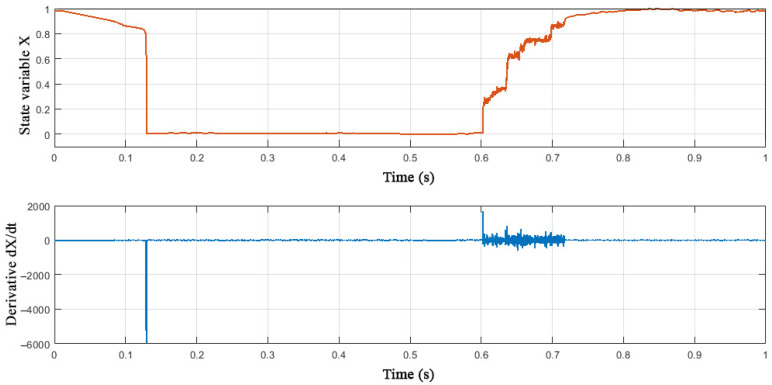
Experimental values of the state variable *X* and its derivative.

At step A1.3.5, numerical differentiation of the state variable *X* is performed to subsequently estimate the time characteristics of the memristor. Figure 15 shows the time series of the state variable *X* and the derivative d*X*/d*t* obtained in steps A1.3.4 and A1.3.5.

**Figure 16:**
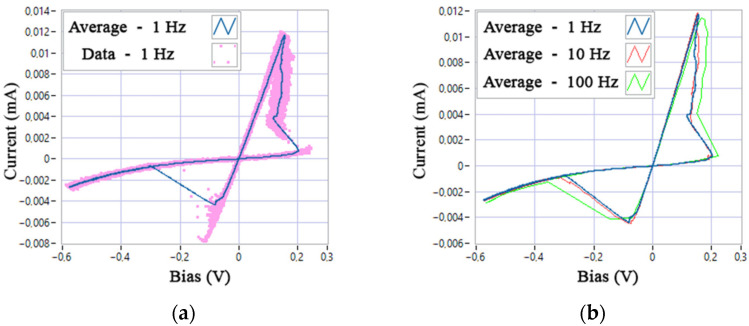
Averaging of experimental I-V curves: (**a**) average cycle and data of a sample of cycles at a frequency of 1 Hz; (**b**) average cycles at different frequencies.

Stage A1.3.2 can be performed in parallel to the previously described stages. At this stage, the I-V curve is filtered and averaged (Figure 16). The averaged I-V curves are then used to determine the functions of the threshold voltages *V*_ON,OFF_(*X*, *F*) of process A1.3.6.

**Figure 17:**
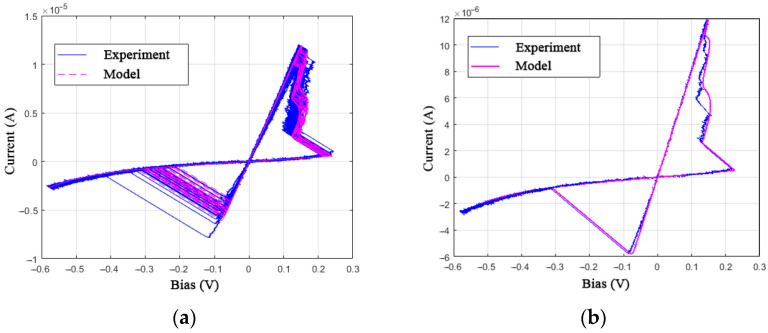
Comparison of the I-V curve of the experimental data and the device model at a frequency of 1 Hz: (**a**) sample and (**b**) a single cycle.

When choosing a chaotic representation of the switching variability, the dependence of the *V*_ON,OFF_ functions from the generator state variables *Y* and *Z* is determined based on the statistical characteristics of the experimental sample (data at a frequency of 1 Hz are shown in Figure 16a and Figure 17a). If one wants to achieve greater model accuracy, it is necessary to transform the distributions of the pseudo-random values *Y* and *Z* of the chaotic generator into an experiment-specific distribution. The solution of this problem by the example of the Zaslavsky web map is given in [47].

In the case of choosing a stochastic representation of the variability in the model, the problem is reduced to simulating the experimental distribution through the application of noise generators.

The final stage of parametric identification is the determination of the time parameters of the model *τ* and *β* in the process A1.3.7, which can be performed by the optimization method of combining one cycle of the experimental and simulated I-V curve (presented in the work of B. Garda and Z. Galias [16] at relative threshold switching voltage values, as shown in Figure 17b. In the same way, but at different frequencies of the control voltage, parameter *τ* must be refined, taking into account the angle *α* of deviation of the current jump-lines from the load *R*_S_ (see Figure 10).

## 4. Discussion

**Figure 18:**
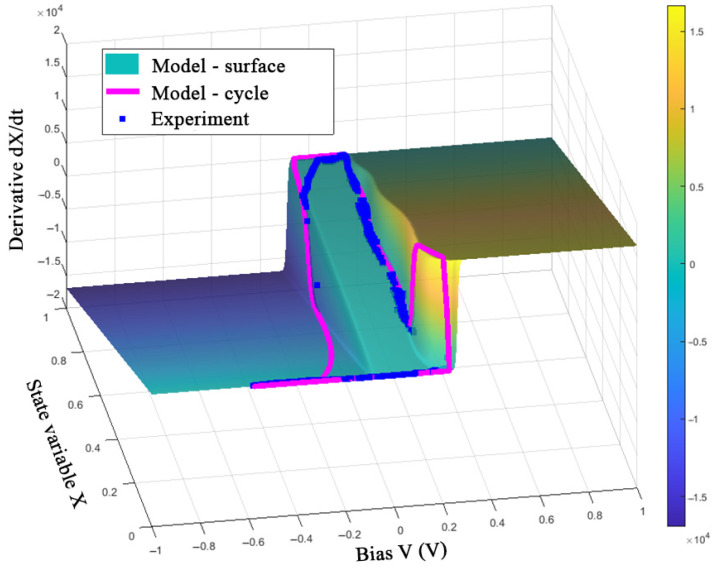
Visualization of the model (11) and experimental data of the device at a frequency of 1 Hz.

**Figure 19:**
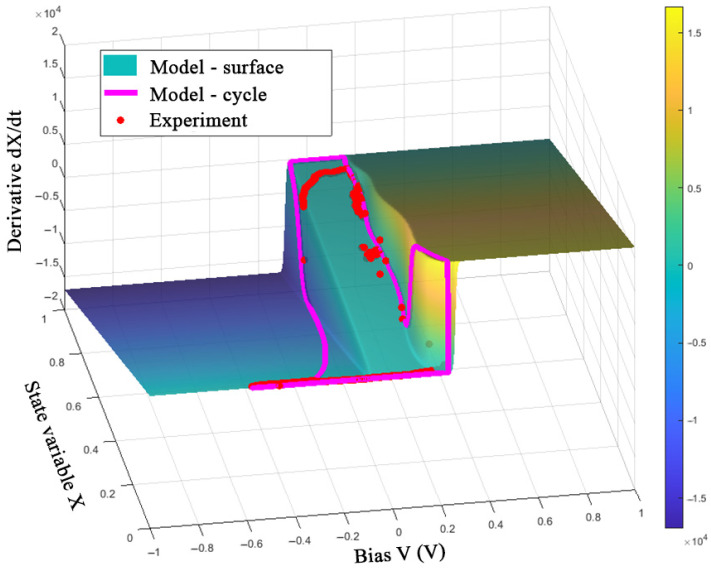
Visualization of the model (11) and experimental data of the device at a frequency of 10 Hz.

**Figure 20:**
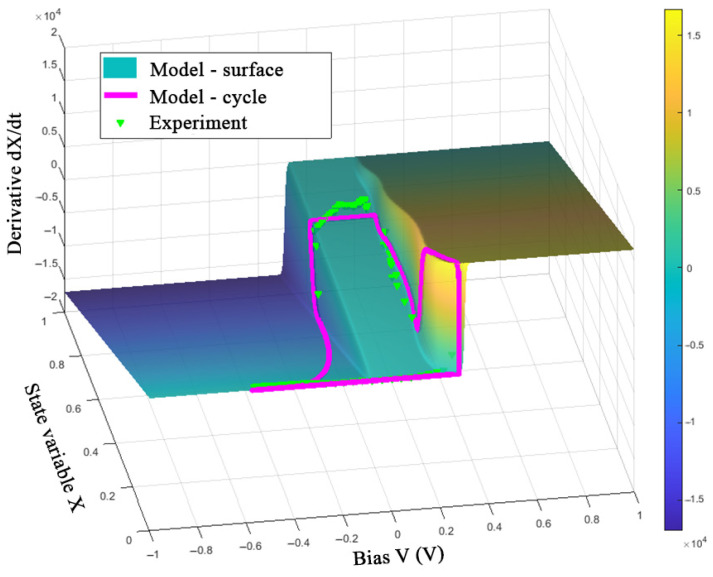
Visualization of the model (11) and experimental data of the device at a frequency of 100 Hz.

The obtained model can be evaluated by representing one switching cycle in the *V*-*X*-d*X*/d*t* space (Figure 18, Figure 19 and Figure 20).

Figure 18, Figure 19 and Figure 20 show the good correspondence between the model dynamics and the experimental data in the processes of switching SET and RESET of the memristor, which indicates the correct selection of the parameters *τ* and *β*. Due to taking into account only the first current jump in the SET process, when using the *V*_ON_ function in the form of (8) under conditions of variable switching, one can see the deviations of the LRS values of variable *X* at frequencies of 10 and 100 Hz. In addition, the deviation of the experimental data points along the *X*-axis from the constant value of the model’s trajectory at negative voltage before the RESET switching is noticeable, which can also be seen in the third quarter of the I-V curve in Figure 17b. This observation can be explained by an error in describing the relationship between the ionic and electronic currents of the device by Equations (6) and (7).

The visible disadvantages in the accuracy of the proposed modified model represented by the system of Equation (11) can be eliminated by complicating the mathematical description of the SET switching process, which takes into account the set of current jumps and their variability, as well as the transcendental Equation (7), taking into account the observed LRS deviation. However, these improvements can lead to a violation of criterion 7-compact representation of a continuous mathematical model, which in turn may mean the impossibility of executing a discrete model on an emulator’s target hardware platform due to its technical limitations.

When discussing potential applications of memristive elements, the focus was made on artificial neural networks. In addition to modeling synaptic connections between neurons, memristors can also be used as intrinsic elements of a spiking neuron modeling ionic conductive channels. A representative example of such a study is [48], where a memcapacitor-based circuit is used to emulate neuronal functionalities. Another necessary element of the presented circuit is a negative differential resistor implemented by VO_2_ device separate from Ag-doped TiO_2-x_-based memcapacitive device. Accounting for the snapback effect in our memristor model allows us to combine the features of negative differential resistance and resistive-switching in a single device, which can improve the scalability and reduce the power consumption of an artificial neuron. Consideration of variability between switching cycles as a chaotic process may also allow us to achieve greater similarity of simulations with the irregular firing of biological neurons. Note that in the case of artificial neural network development, during the memristor identification process one is required to use an extended set of criteria [49], which also includes the learning properties of memristive elements.

## 5. Conclusions

In this paper, we presented a new memristor identification technique in the form of a research-based design process. The steps of the proposed method are demonstrated by the example of modifying the metastable switching memristor models to satisfy the selected set of criteria. In the general case, the process of structural identification is aimed at achieving the temporal, frequential, and statistical characteristics of memristive elements at a qualitative level. The result of the application of the technique was the creation of a new improved mathematical model of the Knowm memristor, using the adaptation of voltage threshold values for the snapback effect, dynamical estimation of the control signal frequency, and the chaotic generator to implement cycle-to-cycle variation. The process of determining the parameters of this model is described inside the framework of parametric identification.

As further research, we plan to apply the proposed chaotic memristor model to develop FPGA-based hardware memristor emulators. Due to this, identification approaches following the adaptive synchronization of the emulator and a real memristive device will be studied.

## Data Availability

Not applicable.
